# A Cross-Sectional and Longitudinal Study to Define Alarmins and A-SAA Variants as Companion Markers in Early Rheumatoid Arthritis

**DOI:** 10.3389/fimmu.2021.638814

**Published:** 2021-08-20

**Authors:** Federica Ciregia, Gwenaël Nys, Gaël Cobraiville, Valérie Badot, Silvana Di Romana, Paschalis Sidiras, Tatiana Sokolova, Patrick Durez, Marianne Fillet, Michel G. Malaise, Dominique de Seny

**Affiliations:** ^1^Laboratory of Rheumatology, University of Liège, Centre Hospitalier Universitaire (CHU) de Liège, Liège, Belgium; ^2^Laboratory for the Analysis of Medicines, Centre Interdisciplinaire De Recherche Sur Le Médicament (CIRM), Department of Pharmacy, University of Liège, Liège, Belgium; ^3^Department of Rheumatology, Centre Hospitalier Universitaire (CHU) Brugmann, Bruxelles, Belgium; ^4^Department of Rheumatology, Centre Hospitalier Universitaire (CHU) Saint–Pierre, Bruxelles, Belgium; ^5^Department of Rheumatology, Hôpital Erasme, Université Libre de Bruxelles, Bruxelles, Belgium; ^6^Department of Rheumatology, Cliniques Universitaires Saint–Luc, Institut de Recherche Expérimentale et Clinique (IREC), Université Catholique de Louvain, Bruxelles, Belgium

**Keywords:** early RA, A-SAA variants, alarmins, DMARDs, companion markers

## Abstract

Nowadays, in the study of rheumatoid arthritis (RA), more and more interest is directed towards an earlier effective therapeutic intervention and the determination of companion markers for predicting response to therapy with the goal to prevent progressive joint damage, deformities, and functional disability. With the present work, we aimed at quantifying in a cohort of early RA (ERA) patients naïve to DMARD therapy, proteins whose increase was previously found associated with RA: serum amyloid A (A-SAA) and alarmins. Five A-SAA variants (SAA1α, SAA1β, SAA1γ, SAA2α, and SAA2β) but also S100A8 and S100A9 proteins were simultaneously quantified in plasma applying a method based on single targeted bottom-up proteomics LC-MS/MS. First, we compared their expression between ERA (n = 100) and healthy subjects (n = 100), then we focused on their trend by monitoring ERA patients naïve to DMARD treatment, 1 year after starting therapy. Only SAA1α and SAA2α levels were increased in ERA patients, and SAA2α appears to mostly mediate the pathological role of A-SAA. Levels of these variants, together with SAA1β, only decreased under biologic DMARD treatment but not under methotrexate monotherapy. This study highlights the importance to better understand the modulation of expression of these variants in ERA in order to subsequently better characterize their biological function. On the other hand, alarmin expression increased in ERA compared to controls but remained elevated after 12 months of methotrexate or biologic treatment. The work overcomes the concept of considering these proteins as biomarkers for diagnosis, demonstrating that SAA1α, SAA1β, and SAA2α variants but also S100A8 and S100A9 do not respond to all early treatment in ERA and should be rather considered as companion markers useful to improve the follow-up of treatment response and remission state. Moreover, it suggests that earlier use of biologics in addition to methotrexate may be worth considering.

## Introduction

Rheumatoid arthritis (RA) is an autoimmune disease characterized by chronic inflammation of synovial joints. In case of severe outcome, the disease results in joint destruction and permanent disability (1–3). Even if many advances have been made in the understanding of RA immunopathology, its etiology still remains not fully elucidated and the prognosis is highly variable. An early recognition of RA can ensure a swift start of the appropriate drug therapy (4, 5). It has been proposed that, ideally, RA diagnosis should be made in the first 12 weeks of manifestation (6). This would result in a better health outcome of patients, promoting higher chance to achieve remission and preserve joint functionality in regard to a longer delay in assessment (6). Therefore, in the last years, the study of RA has moved towards the early phases of the disease.

Moreover, in the ordinary clinical practice, RA patient assessment is based on the disease activity score (DAS28). Despite its widespread use, the main limitation of this scoring system is the inaccuracy in detecting joint inflammation in patients considered as in remission but who may still have joints destruction (7–9). So, the definition of potential companion biomarkers associated to prognosis and therapeutic response could be useful in better characterizing RA disease activity. Among them, the over-expression of two classes of proteins has been extensively associated with RA: serum amyloid A (SAA) and S100 proteins (S100A8, S100A9) (10). For the time being, A-SAA and alarmins have often been considered for diagnostic purpose, but they seem to be quite generic inflammatory markers. Indeed, we want to move forward by rather examining A-SAA variants and S100 proteins in monitoring RA activity and predicting effectiveness of a given therapy in the first year of treatment.

SAA is an acute-phase protein mainly released by hepatocytes, and its concentration rises in trauma, cancer, infection, and inflammatory disease such as RA (11–15). SAA has also been suggested as an indicator of RA activity, considering its correlation with the disease activity score (15–18).

However, less is known about the different role of SAA variants in RA. In humans, there are four different genes encoding SAA: *SAA1*, *SAA2*, *SAA3*, and *SAA4*. *SAA3* was initially referred to as a pseudogene but then its expression was demonstrated in mammary epithelial cell (19), while *SAA4* is constitutively expressed. The so-called acute-phase SAA proteins (A-SAA) are encoded by *SAA1* and *SAA2* genes, which are induced during acute-phase response. The functional role of A-SAA is now debating considering the growing criticism towards studies using recombinant human SAA (rhSAA), a hybrid form of SAA1 and SAA2, contaminated with bacterial lipopolysaccharides and lipoproteins and therefore enhancing TLR2 and TLR4 pathways (20, 21). It highlights the inconsistency among endogenous A-SAA and rhSAA activity (22, 23), as well as the importance of redefining pro-inflammatory properties of A-SAA. Recently, researchers discovered that SAA1 and SAA2 promote differentiation of pathogenic Th17 cells (24) using loss- and gain-of-function mouse models and that SAA1 initiates type 2 immunity taking part to inflammatory disease (25). It has also been shown that rhSAA1, free of any bacterial contaminants, lacked the previously reported TLR2-mediated activities but preserved its role in neutrophil chemotaxis in synergy with CXCL8 *via* FPR2 (26).

Another issue in the study of A-SAA is the simultaneous presence of different isoforms Kim et al. showed in lung cancer differences in the expression of each subtype (27). Indeed, *SAA1* and *SAA2* are polymorphic with three and two variants, respectively: SAA1α, SAA1β, SAA1γ, SAA2α, and SAA2β. The difficulty in the identification of these different variants consists in their high homology (>90%). Since they only differ in few amino acids, commercial antibodies usually react with all isoforms. Recently, we developed a method to quantify simultaneously each of these variants by single targeted bottom-up proteomics LC-MS/MS (28). The expression of these different isoforms was investigated in various immune-mediated inflammatory diseases (IMIDs: RA, ankylosing spondylitis, systemic lupus erythematous, systemic sclerosis, osteoarthritis) highlighting quantitative as well as qualitative differences among A-SAA variants. Moreover, a negative correlation was determined between SAA1α and SAA1β levels, and a mirror symmetry was observed between both levels throughout all IMIDs (28). In addition to A-SAA variants, our method quantified also S100A8 and S100A9 proteins. S100A8 and S100A9 alarmins also known as myeloid-related protein (MRP)-8 and MRP-14, or calgranulin A and B, respectively, are two members of the S100 protein family which are expressed in monocytes, granulocytes, and neutrophils to modulate inflammatory response (29). The extracellular heterodimer they formed is called calprotectin, and it is routinely measured in gastrointestinal inflammation (30, 31). However, high levels of S100A8 and A9 have also been detected in many immune system dysfunction diseases such as psoriatic arthritis, systemic lupus erythematosus, ankylosing spondylitis, and RA (28, 32–34). In RA, neutrophils are strong producers of the S100A8/A9 alarmins whose presence is linked to joint erosion *via* the induction of inflammation (35–37).

The aim of the present study was to characterize the expression of the five different A-SAA variants (SAA1α, SAA1β, SAA1γ, SAA2α, and SAA2β) and S100A8/A9 proteins in a large national cohort of early RA (ERA) patients, in regard to healthy control subjects. Proteins levels were quantified employing the method we previously developed by LC-MS/MS (28). Treatment-naïve patients were recruited after their first medical examination and subsequently after 12 months of treatment in order to investigate the therapeutic effect on A-SAA and S100 protein expression.

## Material and Methods

### Patients

One hundred patients (80 females, 20 males) suffering from ERA (mean age 34.5 ± 9.9; M ± SD) and 100 healthy controls, well matched for age (34.4 ± 8.9) and sex (80 females, 20 males), were enrolled in the study. All patients fulfilled the 2010 ACR/EULAR disease classification criteria (38). The cohort of ERA included patients younger than 50 years old, with a disease duration <3 months and naïve to DMARD therapy at time 0 (T0). Each patient was assessed for DAS28 using C-reactive protein (DAS28-CRP), clinical disease activity index (CDAI), and simplified disease activity index (SDAI) at baseline and during a 1-year follow-up. The response to therapy takes into account the modification of DAS28-CRP after treatment so that it can be evaluated as good, moderate, or absent according to the EULAR response criteria (39). In our study, ERA patients were sorted in good responders (R; n = 48) and non-responders (NR; n = 48). The response was considered good when the DAS28-CRP at T12 was <3.2 and decreased by a factor of at least 1.2 from baseline (T0) (39). All the other patients belonged to NR. Four patients were removed from the analysis because they did not fulfill the criteria for either R or NR. Human blood samples were further collected from patients after 1 year of treatment (T12), in order to evaluate the response to treatment. **Table 1** summarizes the clinical data of participants who were included in the study.

**Table 1:** Clinical characteristics of subjects.

	HV	ERA	
n	100	100	
Female	80	80	
Age - Mean *(range)*	34.4 *(19–51)*	34.5 *(16–50)*	
RF+ %	–	64	
Anti-CCP+ %	–	65	
**Treatment at T12%**			
Corticosteroids	–	11	
Methotrexate	–	88	
Biologics*	–	23	
Hydroxychloroquine	–	5	
**Clinical measures**		**Mean *(range)* T0**	**Mean *(range)* T12**
DAS28-CRP	–	4.4 *(1.21–7.27)*	2.8 *(1.21–5.9)*
CRP mg/dl	–	2.2 *(0.09–26)*	0.5 *(0.02–10)*
SDAI	–	23.6 *(0–70)*	10 *(0.1–77)*
CDAI	–	21.5 *(0–65)*	8.7 *(0–55)*
TJC	–	9.4 *(0–33)*	3.5 *(0–45)*
TJC 28	–	7 *(0–26)*	2.5 *(0–23)*
SJC	–	6.4 *(0–27)*	1.6 *(0–19)*
SJC 28	–	4.9 *(0–26)*	1.3 *(0–19)*
HAQ	–	1 *(0–2.8)*	0.7 *(0–3.8)*
VAS *medical*	–	42.4 *(5–89)*	16 *(0–70)*
VAS *patient*	–	54.1 *(0–100)*	33.4 *(0–90)*
VAS *pain*	–	54.4 *(0–100)*	32 *(0–90)*
VAS *fatigue*	–	55.5 *(0–100)*	42.5 *(0–96)*

RF, rheumatoid factor; anti-CCP, anti-cyclic citrullinated peptide; DAS28–CRP, Disease Activity Score 28 joints; CRP, C-reactive protein; SDAI, Simplified Disease Activity Index; CDAI, Clinical Disease Activity Index; TJC 28: 28-Tender Joint Count; SJC 28, 28-Swollen Joint Count; HAQ, Health Assessment Questionnaire; VAS, Visual Analogue Scale.

*15% anti-IL-6; 15% anti-JAK1/JAK2; 70% anti-TNFα.

Clinical parameters of 100 ERA patients enrolled in the study at time 0 (T0) and after 12 months of treatment (T12) and compared to 100 healthy volunteers (HV). ERA, early rheumatoid arthritis.

#### Ethics

An informed consent was obtained from all recruited subjects and the study was approved by Ethics Committee of the Cliniques Universitaires Saint-Luc (Bruxelles; Study No. B403201317717).

### Sample Collection

Human blood samples were collected in standard conditions and allowed to coagulate in plain glass tubes or EDTA-treated tubes to isolate serum or plasma, respectively. Serum and plasma were obtained after centrifugation at 2000 x*g* for 10 min, room temperature. Supernatants were aliquoted and stored at −80°C until use.

### Quantitation of A-SAA Variants and S100 Proteins

To quantify simultaneously in plasma the five different A-SAA variants, S100A8 and S100A9 proteins, we applied a method that has been recently developed in our laboratory (28): targeted bottom-up proteomics LC-MS/MS. Briefly, internal standard (ISTD) as well as all standard peptides and proteins for S100 and SAA were dissolved in H_2_O/ACN/FA (90:10:0.1, v/v), aliquoted, and stored at −80°C until use. A mix of calibrant solution and bovine plasma was loaded on a 96 plate in duplicate; concurrently, the samples were mixed with H_2_O/ACN/FA (80:20:0.1, v/v). Then, ammonium bicarbonate solution containing 33.3% of MeOH and ISTD was added in each well. Finally, after 10 min of incubation at 100°C, trypsin solution was added overnight at 37°C. Ammonia was added to quench the reaction. The Oasis Max SPE plate of 10 mg (Waters Corporation, Dublin, Ireland) has been used for extraction. For LC-MS analysis, ultra-high-pressure liquid chromatography separation was performed using a 1290 infinity system (Agilent Technologies, Waldbronn, Germany) with a C18 column (Phenomenex, Torrance, CA, USA). The separation was performed by gradient mode. MS/MS detection was achieved on a 6495 LC-MS TripleQuadrupole supplied with the iFunnel Technology (Agilent Technologies) and operated using positive electrospray ionization.

### ELISA

The concentration of total A-SAA and calprotectin was detected in serum by commercial ELISA kits (A-SAA from Thermo Fisher Scientific, Waltham, MA, USA; calprotectin from Bühlmann, Schönenbuch, Switzerland) used according to the manufacturer’s instructions. The calibration range was from 9.4 to 600 ng/ml and 4 to 240 ng/ml for A-SAA and calprotectin, respectively. Serum was diluted 1:1000 for A-SAA and 1:100 for calprotectin. All experiments were performed in duplicate.

### Statistics

Statistical analysis was performed with SPSS (SPSS/PC Statistical Package for the Social Science, update for 10.1. Chicago, IL: SPSS Inc., 2000), Graph Pad Prism 6 software, and G*Power 3.1 software.

#### Comparisons

Concentrations (ng/ml) obtained from LC-MS/MS and ELISA are presented after a logarithmic transformation (base 10), and to determine whether data were normally distributed, a D’Agostino-Pearson test was applied. Comparisons between groups were performed using non-parametric tests. Analysis of control *vs.* T0 or T12 was made with the Kolmogorov–Smirnov test for unpaired data, and Wilcoxon signed rank test was applied for paired data between T0 and T12. Differences were considered as statistically significant when p-value was ≤0.05.

#### Power Analysis

We performed a *post hoc* power analysis to determine if the sample size was appropriate. The power analysis was based on the measure of the effect size, through Cohen coefficient calculation (40), and was performed for all statistically significant comparisons. We considered that the sample size was correct when it allowed to reach a level of power ≥ 0.95.

#### Correlation

To evaluate the statistical correlation among proteins and clinical measures, Spearman’s rank correlation coefficient was calculated.

#### ROC Curves

Receiver operating characteristic (ROC) curves were plotted to determine marker performance in discriminating ERA patients from controls. It was also estimated whether a combination of different proteins might increase this performance. Hence, a logistic regression was applied to calculate the weight given by each marker and to determine the formula for having a combined risk index. In order to evaluate whether the marker combination might increase the performance in distinguishing ERA from healthy subjects, area under curve (AUC) was calculated with 95% confidence interval, evaluating sensitivity and specificity of each marker and their combination.

## Results

### Classification of Patients

**Figure 1:**
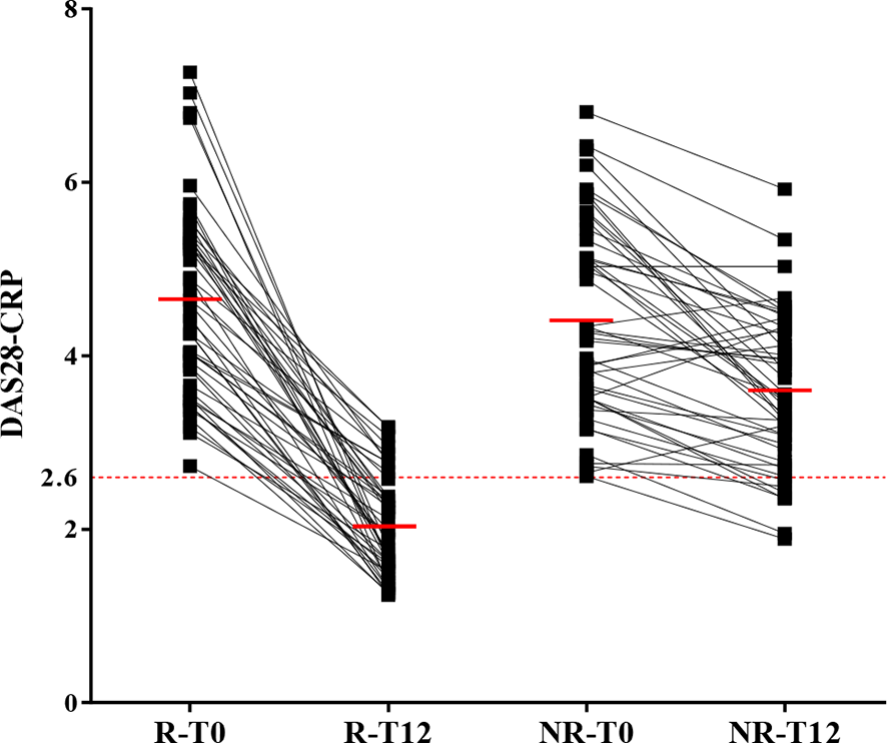
Classification of ERA patients in good and non-responders according to DAS28-CRP. The 100 patients suffering from ERA were divided in two groups: good (R) and non-responders (NR) according to the variation of DAS28-CRP from T0 and T12 (after 12 months of treatment). When DAS28-CRP ≤ 2.6, the patient is in remission (dotted red line). The mean for each group is indicated with a full red line. Four patients were removed from the analysis because they did not fulfill the criteria for either R or NR.

In our cohort, we had 48 R and 48 NR. Patients of the R group had a mean DAS28-CRP value of 4.6 at T0 decreasing to 2 at T12. In the NR group, the mean DAS28-CRP value was of 4.4 at T0 and 3.6 at T12 (**Figure 1**). The main treatment was methotrexate (88%) followed by biologics (23%), corticosteroids (11%), and hydroxychloroquine (5%).

### Quantification of A-SAA

The expression of five A-SAA variants was quantified in plasma of 100 ERA patients and 100 healthy controls by LC-MS/MS. Comparisons were considered as statistically significant when p-value was ≤0.05 and power of sample size was ≥0.95.

**Figure 2:**
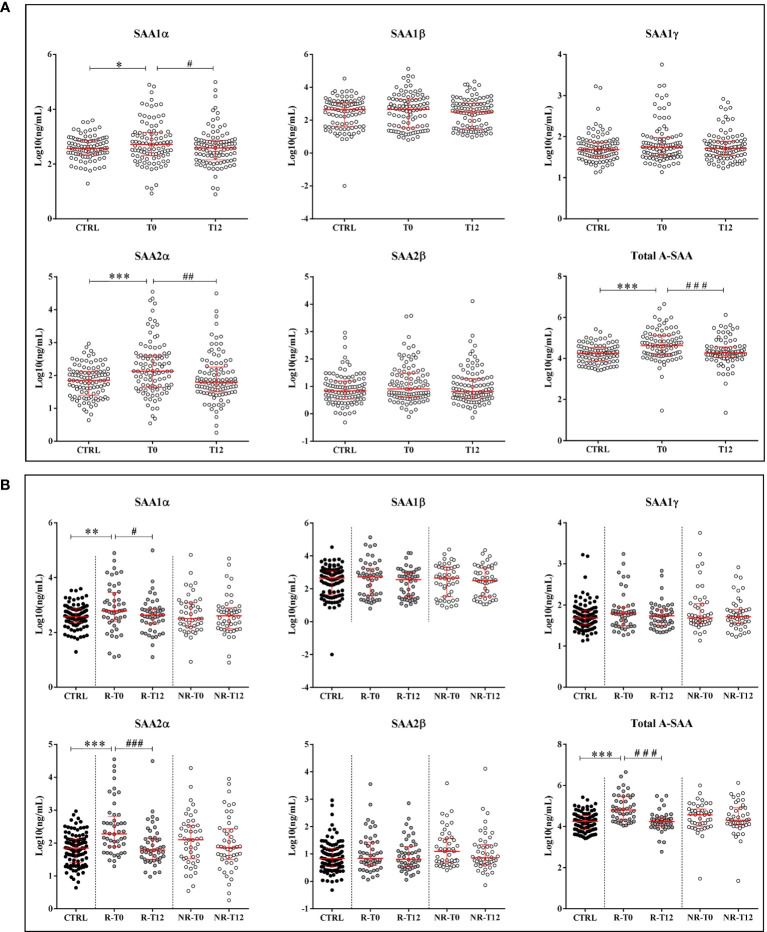
Quantification of A-SAA by LC-MS/MS and ELISA. **(A)** Expression of A-SAA variants and total A-SAA in healthy volunteers (controls, CTRL) and ERA patients at time T0 and after 12 months of treatment (T12). **(B)** Expression of A-SAA variants and total A-SAA in ERA patients sorted in two groups: good (R) and non-responders (NR). Scatter dot plots represent the median with interquartile range. *p-value ≤ 0.05, **p-value ≤ 0.01, ***p-value ≤ 0.001 (Kolmogorov–Smirnov test); ^#^p-value ≤ 0.05, ^##^p-value ≤ 0.01; ^###^p-value ≤ 0.001 (Wilcoxon test).

In ERA patients at T0 compared to healthy subjects, we found that the concentration of SAA1α and SAA2α variants was significantly increased with a p-value of 0.02 and <0.001, respectively (**Figure 2A**). When comparing T0 *vs.* T12, a significant decrease was observed after therapy for SAA1α (p-value = 0.02) and SAA2α (p-value = 0.003), suggesting that these two variants are good responders to treatment (**Figure 2A**). Subsequently, we examined the variation in the R and NR groups (**Figure 2B**) for all A-SAA variants. It is interesting to observe that the significant difference was only present in the group of good responders. Indeed, when comparing ERA patients at T0 *vs.* controls, we observed a significant increase for SAA1α (p-values = 0.005) and SAA2α (p-value < 0.001) only in the R group (**Figure 2B**). Similarly, the decrease at T12 compared to T0 was also only significant in the R group for SAA1α and SAA2α with p-values of 0.01 and <0.001, respectively.

In addition, by measuring the relative proportion of each variant, we observed that the contribution of SAA1α and SAA1β was higher compared to the others, and in ERA patients, the proportion of SAA1β and SAA2α showed a tendency to decrease and increase, respectively, compared to controls (**Supplementary Figure S1**).

Finally, we quantified by ELISA the expression of total A-SAA (**Figures 2A, B**) and also observed in the R group a significant increase of A-SAA for ERA patients at T0 *vs.* controls (p-value < 0.001) and a decrease at T12 *vs.* T0 (p-value < 0.001). We also found a significant correlation among ELISA results and the expression of SAA1α, SAA1β, and SAA2α (**Table 2**).

**Table 2:** Statistical correlation among proteins and clinical measures.

		1α	1β	1γ	2α	2β	A-SAA	S100A8	S100A9	CALP
**1α**	*a*	–	-0.28	n.s.	0.581	n.s.	0.296	0.274	0.376	n.s.
	*b*		0.005		3e^-10^		0.005	0.006	1e^-04^	
**1β**	*a*	-0.28	-	0.212	0.200	0.252	0.480	n.s.	n.s.	0.244
	*b*	0.005		0.036	0.049	0.012	2e^-06^			0.022
**1γ**	*a*	n.s.	0.212	–	n.s.	0.213	n.s.	0.250	n.s.	n.s.
	*b*		0.036			0.034		0.012		
**2α**	*a*	0.581	0.200	n.s.	-	n.s.	0.655	0.393	0.517	0.393
	*b*	3e^-10^	0.049				2e^-12^	5e^-05^	3e^-08^	1e^-04^
**2β**	*a*	n.s.	0.252	0.213	n.s.	–	n.s.	n.s.	0.250	n.s.
	*b*		0.012	0.034					0.012	
**S100A8**	*a*	0.274	n.s.	0.250	0.393	n.s.	0.276	-	0.681	0.433
	*b*	0.006		0.012	5e^-05^		0.008		7e^-15^	2e^-0^5
**S100A9**	*a*	0.376	n.s.	n.s.	0.517	0.250	0.427	0.681	–	0.434
	*b*	1e^-04^			3e^-08^	0.012	3e^-05^	7e^-15^		2e^-05^
**DAS28**	*a*	0.239	n.s.	n.s.	0.300	n.s.	0.208	n.s.	n.s.	0.309
	*b*	0.017			0.002		0.049			0.003
**SDAI**	*a*	0.225	n.s.	n.s.	0.238	n.s.	n.s.	n.s.	n.s.	0.251
	*b*	0.025			0.017					0.017
**CDAI**	*a*	n.s.	n.s.	n.s.	n.s.	n.s.	n.s.	n.s.	n.s.	n.s.
	*b*									
**TJC**	*a*	n.s.	n.s.	n.s.	n.s.	n.s.	n.s.	n.s.	n.s.	n.s.
	*b*									
**TJC28**	*a*	n.s.	n.s.	n.s.	n.s.	n.s.	n.s.	n.s.	n.s.	n.s.
	*b*									
**SJC**	*a*	0.264	n.s.	n.s.	0.226	n.s.	n.s.	n.s.	n.s.	n.s.
	*b*	0.009			0.025					
**SJC28**	*a*	n.s.	n.s.	n.s.	n.s.	n.s.	n.s.	n.s.	n.s.	n.s.
	*b*									
**CRP mg/dl**	*a*	0.293	n.s.	n.s.	0.415	n.s.	0.450	n.s.	0.263	0.292
	*b*	0.003			2e^-05^		8e^-06^		0.008	0.005
**HAQ**	*a*	n.s.	n.s.	n.s.	0.250	n.s.	n.s.	n.s.	n.s.	n.s.
	*b*				0.012					
**VAS medical**	*a*	0.250	n.s.	n.s.	0.240	n.s.	n.s.	n.s.	n.s.	n.s.
	*b*	0.013			0.016					
**VAS patient**	*a*	n.s.	n.s.	n.s.	0.238	n.s.	n.s.	n.s.	n.s.	0.343
	*b*				0.017					0.001
**VAS pain**	*a*	n.s.	n.s.	n.s.	0.207	0.227	n.s.	n.s.	n.s.	0.220
	*b*				0.041	0.025				0.039
**VAS fatigue**	*a*	n.s.	n.s.	n.s.	n.s.	n.s.	n.s.	n.s.	n.s.	0.241
	*b*									0.029

a, correlation coefficient; b, p-value; n.s., not significant.

The clinical correlations among A-SAA variants and S100 proteins with clinical features (at time T0) were determined by calculating the Spearman’s rank correlation coefficient.

### Quantification of Alarmins

**Figure 3:**
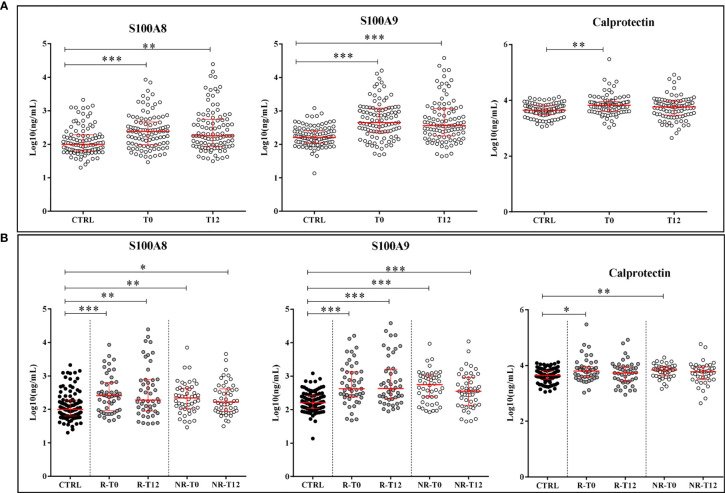
Quantification of proteins S100A8, S100A9, and calprotectin. **(A)** Expression of alarmins in controls (CTRL) and ERA patients at time T0 and after 12 months of treatment (T12). **(B)** Variation of the expression of alarmins in good (R) and non-responders (NR). Scatter dot plots represent the median with interquartile range. *p-value ≤ 0.05, **p-value ≤ 0.01, ***p-value ≤ 0.001 (Kolmogorov–Smirnov test).

Simultaneously to A-SAA variants, the LC-MS/MS method allowed to quantify S100A8 and S100A9 proteins. As expected, their expression was significantly increased in plasma of ERA patients (T0) *vs.* healthy subjects with a p-value < 0.0001 (**Figure 3A**). In contrast to A-SAA, we did not observe any significant decrease at T12, after treatment. Indeed, the intensity of S100A8 and S100A9 was still significantly increased compared to controls with a p-value of 0.004 and <0.0001, respectively, and with no variation between T0 and T12. That remained unchanged when patients were reclassified in the R and NR groups. There was no difference between R and NR at T0, or either at T12. The level of both proteins still remained significantly higher for ERA patients *vs.* healthy subjects, at T0 and T12, for R and NR classes (**Figure 3B**). Thereafter, the presence of calprotectin in serum was quantified. **Figure 3A** shows that the expression of calprotectin in ERA was increased compared to controls (p-value = 0.0011). While calprotectin at T0 compared with controls increased in the R and NR classes (with a p-value of 0.04 and 0.0011, respectively), no difference was observed between R and NR at T0, or either at T12 (**Figure 3B**).

### Clinical Correlations

Correlation parameters among protein intensities and clinical features were determined. Results are summarized in **Table 2**.

#### A-SAA Variants

Only total A-SAA, SAA1α, and SAA2α showed a significant positive correlation with DAS28-CRP, which is in accordance with differences observed between the R and NR groups as described above for A-SAA variants. It is worth to mention that SAA1α and SAA2α variants were statistically more correlated with functional parameters in regard to total A-SAA. SAA1α was correlated with SDAI, SJC, and VAS medical, whereas SAA2α, besides these, also correlated with HAQ, VAS patient, and VAS pain (**Table 2**).

#### Alarmins

The expressions of S100A8 and S100A9 were strictly correlated between themselves but not to other clinical parameters. They did not show, as expected, any correlation with DAS28-CRP, which is coherent with the absence of variation observed for these proteins in response to treatment. Interestingly, when the correlation between S100A8 and S100A9 was evaluated, a better correlation of their expression was observed in ERA (T0) compared to healthy subjects (**Supplementary Figure S2A**). Same result was obtained when calprotectin was correlated with S100A8 and S100A9: correlation was only observed with ERA patients but not with controls (**Supplementary Figures S2B, C**). Moreover, if calprotectin is correlated with SDAI and VAS (patient, pain, fatigue), the single alarmins did not show any correlation with clinical parameters (**Table 2**).

### ROC Curves

In order to define the clinical potential of selected proteins to differentiate between ERA patients and healthy controls, ROC curves were calculated with A-SAA variants, alarmins, and C-reactive protein (CRP) intensities. In addition, a logistic regression analysis was applied to assess the area under the curve (AUC) of the ROC curves obtained with combined markers, in order to analyze whether the discriminative power might increase. The combinations giving the better ROC curves are presented in **Supplementary Figure S3**.

#### A-SAA Variants

ROC curves were calculated for the five A-SAA variants, but single variant analysis did not provide any significant interest for clinical diagnosis (**Supplementary Figure S4A**). We highlighted that the best curve was obtained with SAA2α variant, which was similar to the one obtained with total A-SAA. Nevertheless, it remains interesting to mention that the best ROC curve was derived from the SAA2α/CRP combination (**Supplementary Figures S3A, C**), while ROC curves including total A-SAA had slightly less diagnostic power (**Supplementary Figures S4B, C**).

#### Alarmins

ROC curves with alarmins were calculated, alone or in combination with other parameters. The best ROC curve was achieved with the combination S100A8/S100A9/CRP (**Supplementary Figure S3B**), which outreached the discriminating power of each single protein (**Supplementary Figure S3C**).

### Expression of Proteins in Response to Treatment

The influence of different DMARDs prescribed for therapy was explored by examining the expression of A-SAA variants and S100 proteins according to patient’s treatment at T12. Therefore, patients treated with methotrexate monotherapy (n = 58 with 24% NR) were separated from patients treated with biological drugs (n = 23 with 13% NR). CRP levels decreased at T12 with both treatments compared to baseline, with a higher effect under biologic therapy (**Supplementary Figure S5A**).

#### A-SAA Variants

**Figure 4:**
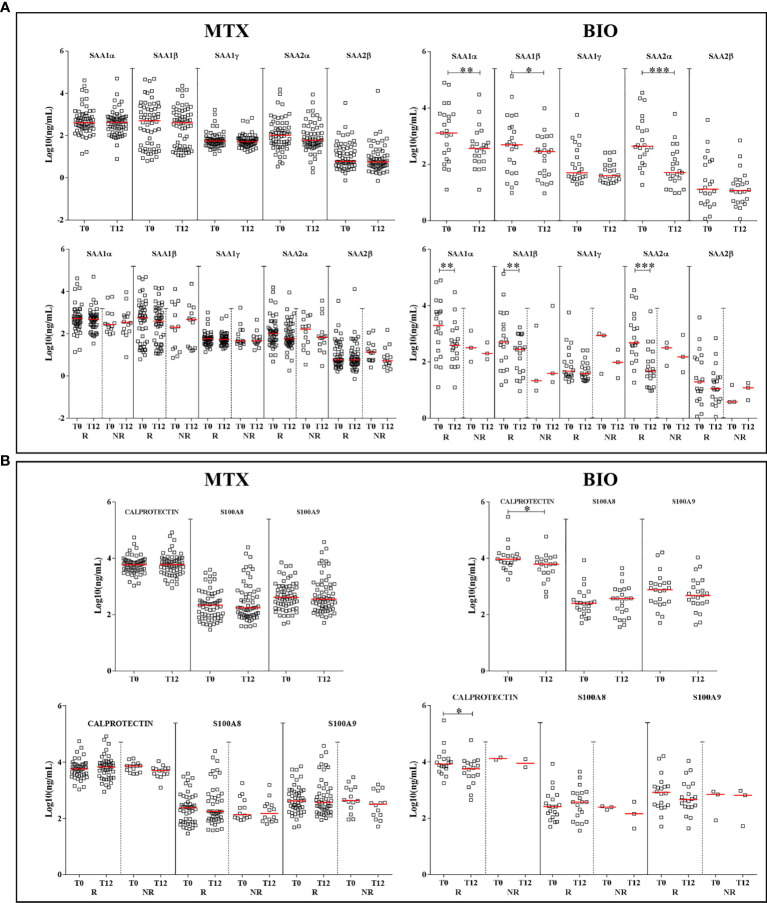
Differences in the expression of proteins according to the treatment. Graphs depict the expression of A-SAA variants **(A)** and alarmins **(B)** in patients, at T0 and T12, sorted by treatment: methotrexate monotherapy (MTX) or biologics (BIO). Scatter dot plots represent the median with interquartile range. *p-value ≤ 0.05, **p-value ≤ 0.01, ***p-value ≤ 0.001 (Wilcoxon test). R, good responders; NR, non-responders.

In the comparison T0 *vs.* T12, there was a significant decrease at T12 for SAA1α, 1β, and 2α with a p-value of 0.0011, 0.03, and <0.0001, respectively, only under biologic antirheumatic. When ERA patients were sorted in R and NR groups, the significant decrease at T12 *vs.* T0 was only observed in the R group with a p-value of 0.002, 0.002, and <0.0001 for SAA1α, 1β, and 2α, respectively (**Figure 4A**). Total A-SAA levels decreased only under biologic therapy (p-value < 0.001) (**Supplementary Figure S5B**). By sorting R and NR patients, the decrease emerged also in the methotrexate group but only for good responders, and it was more pronounced under biologic therapy (**Supplementary Figure S5B**).

#### Alarmins

For alarmins, we did not observe any significant reduction at T12 when patients received methotrexate. A difference was found after biologic therapy for calprotectin in the group of good responders (p-value = 0.01) (**Figure 4B**). However, it is important to point out that the NR group under biologic therapy was too small (n = 3) for a consistent statistical analysis.

## Discussion

In our cohort of 100 ERA patients, of which 88% had methotrexate and 23% received biologics, we only observed the presence of 48 good responders despite 1 year of treatment. Further, in the group of good responders, we still observed the expression of inflammatory markers.

The early therapeutic intervention is crucial in ERA, leading to better long-term prognosis and prevention of structural damage. At this time, antibodies against cyclic citrullinated proteins and rheumatoid factor are used to diagnose ERA in common clinical practice (41). On the other hand, the parameter commonly used to evaluate the disease activity of RA is DAS28-CRP, a composite score based on clinical and laboratory data, specifically CRP. Nevertheless, some limitations in its use have emerged (7–9, 42, 43), so companion markers take up a key role in improving the predictability of patient’s response giving a tool for helping treatment’s choice. Some proteins have been introduced (41) to gradually unveil RA pathophysiological mechanisms and response to treatment such as A-SAA and alarmins. These are typical inflammation biomarkers whose increase has been widely reported in RA. However, in this study, we explored the expression level of A-SAA variants in ERA, which has been less described, and of alarmins that have been mainly studied as the heterodimeric calprotectin in serum, but not as monomeric proteins in plasma as recommended by Nordal et al. (44) Above all, we focused on their trend to decrease from baseline in DMARDs-naïve patients after 1 year of treatment, exploring their usefulness as companion markers for monitoring RA activity and the inflammatory process. This cross-sectional and longitudinal study belongs to the early phase of markers validation. Other studies will be necessary to assess their analytical and clinical validation.

A-SAA is progressively being considered as a more reliable factor than CRP in detecting subclinical inflammation. Indeed, A-SAA was shown to have a better association with the disease activity than CRP in inflammatory rheumatic diseases (16, 18, 43, 45, 46). Interestingly, the well-described biological roles of A-SAA have been recently reconsidered. Actually, several studies have now demonstrated the unreliability of using bacterial recombinant form of A-SAA due to (i) its hybrid amino acid composition between SAA1 and SAA2 isoforms and (ii) the presence of bacterial contaminants enhancing pro-inflammatory pathways (18, 21–23, 26). They also substantiated the need of differentiating A-SAA variants from each other, which cannot be performed by an antibody-based approach such as the ELISA kits.

The quantification of the five different A-SAA variants is challenging since they differ in few amino acids (Supplementary Data 1). Lately, we have developed a method by LC-MS/MS which allowed to simultaneously discriminate the five different A-SAA variants (28). This earlier work found a difference in the expression of isoforms related to RA disease activity. Moreover, a negative correlation was observed between SAA1α and SAA1β levels. Hence, this gave us a hint about the clinical relevance of distinguishing these variants in different immune-mediated inflammatory diseases. Therefore, we have now measured A-SAA variants in plasma from ERA patients and healthy controls. Besides LC-MS/MS, total A-SAA was quantified in serum by ELISA. We observed the increase of A-SAA in ERA patients at T0 compared to controls and a new significant decrease after 12 months of therapy for the good responders. This trend was observed by MS for SAA1α and SAA2α expression, the latter isoform presenting the most statistically significant variation. Furthermore, we observed that total A-SAA was correlated with SAA1α, SAA1β, and mainly with SAA2α. These data suggest that the effect of A-SAA could be primarily related to SAA2α. Actually, when ROC curves were plotted for assessing the clinical potential of A-SAA variants, the best curve included SAA2α but not total A-SAA. It does not mean that SAA2α should be considered as a useful biomarker; we can rather state the weak usefulness of A-SAA proteins as biomarkers compared to CRP, but it suggests that it is important to differentiate A-SAA variants when studying the role of A-SAA in chronic inflammatory diseases. Accordingly, it should be highlighted that only SAA1α and SAA2α were correlated with the DAS28 as also observed with A-SAA. But most importantly, unlike total A-SAA, only SAA1α and specifically SAA2α were correlated with several clinical parameters (i.e., SDAI, SJC, HAQ, and VAS). Among these correlations, the one with the SJC parameter is noteworthy as it is directly related to a joint alteration and patients considered as in remission could experience joint damage. So, these observations suggest that not all variants have the same weight in ERA pathophysiology and that single variants can also give more information than total A-SAA.

Then, we examined whether there was a difference in the expression of A-SAA in relation to the type of therapy used at T12. One-year treatments were diversified; nevertheless, we identified two groups of treated ERA patients at T12: a first group receiving methotrexate as monotherapy, and a second group receiving biologic antirheumatic drugs. The group of patients under methotrexate showed a higher percentage of NR than the group using biologic drugs.

As already described (16), ERA patients treated with biologic therapy showed at T12 a higher decrease in A-SAA levels compared to those treated with methotrexate monotherapy. Some authors also assessed that the concentration of A-SAA decreased at the beginning of therapy but that it was followed by a slight A-SAA increase after 12 months of treatment (47). Interestingly, when we focused on the single variants, we observed that the response to treatment was only observed for SAA1α, SAA1β, and SAA2α in good responders under biologic therapy but not after methotrexate monotherapy, unlike CRP and total A-SAA. This suggests that single variants could be more useful to stratify RA patients and to monitor treatment.

The alarmins S100A8 and S100A9 and their corresponding heterodimer, calprotectin, are proteins involved in the inflammatory process in RA (32, 33, 48). Consistent with previous findings (49), we stated a significant increase of these proteins in ERA patients compared to healthy subjects. We also calculated a good ROC curve by combining S100A8/S100A9/CRP, but our study did not include any disease control group, so the observed specificity was not highly relevant. After all, alarmins and A-SAA proteins are well-known ubiquitous markers of inflammation, and cannot replace gold standards in RA diagnosis such as antibodies against cyclic citrullinated proteins and rheumatoid factor. In fact, the substantial observation of our longitudinal study was that alarmin levels remained elevated after 1 year of treatment. There was even no difference between good and non-responders, and also no correlation of S100A8 and S100A9 with DAS28-CRP. Moreover, we also observed that the expression of S100A8 was even more correlated with S100A9 under pathological conditions compared to healthy subjects. The same observation was pointed out when correlating calprotectin with S100A8 and S100A9. This might suggest a different role of S100 for ERA patients compared to controls.

The usefulness of alarmins as companion markers for treatment response has been extensively debated, with various results. This is linked to the wide differences in cohorts and selected therapies for different studies (49–56). In our study, we observed that the group under methotrexate or biologic DMARD treatment did not present any decrease of alarmin expression, except for a small decrease of calprotectin at T12 in the R group receiving biologic drugs. This is in accordance with another study suggesting that calprotectin could not be considered as a predictor of clinical response to methotrexate in ERA (49, 56). Conversely, it has also been proposed that calprotectin can decrease during therapy with biologics agents (50, 51).

Hence, in line with previous observations (47), the outcome of our longitudinal analysis suggests that despite the fact that clinical evaluation classifies a patient as being in remission according to the DAS28-CRP, some inflammatory markers can still remain and the assessment of classical markers might not be sufficient to properly follow the inflammatory process. Indeed, CRP is widely used as indicator of chronic inflammation, also in RA, but it has already been suggested (43) that CRP by itself cannot be used to evaluate the remission status in RA patients. Likely, the systemic inflammation process is not coupled to the radiological evidence of persisting injury (43). Thus, patients can be considered in clinical remission but still having joint inflammation, which could be highlighted by the presence of calprotectin (or S100A8/S100A9 proteins). Actually, calprotectin, unlike CRP, is a protein that during inflammation is mainly produced by macrophages and granulocytes infiltrating the RA synovial membrane (51). Moreover, the presence of S100A8/A9 alarmins is linked to joint erosion in RA (35–37). Therefore, it directly reflects synovial inflammation (52, 55, 56).

In conclusion, in RA treatment, the achievement of clinical remission has the ultimate goal to prevent progressive joint damage, deformities, and functional disability. Anyway, structural destruction can occur independently of arthritis activity assessed by DAS28 (7, 9, 57). In order to overcome this limit, our study highlighted the importance to simultaneously quantify A-SAA variants and alarmins. Indeed, SAA1α, SAA1β, and SAA2α followed the response to therapy after 1 year of biologic DMARD treatment but not under methotrexate, and SAA2α was the variant which appeared to mostly mediate the role of A-SAA. Currently, there are few papers describing these variants whose study is challenging. Our work highlighted the need of differentiating isoforms of A-SAA in the study of RA. Indeed, it will be interesting to characterize their biological role, since some variants are modulated in inflammatory condition and after response to treatment, while some others are not. Therefore, this fundamental and translational research paves the way for future functional studies. Currently, there are no papers describing these variants and their biological role, mainly because of the absence of reliable commercial SAA proteins/variants for *in vitro*/*in vivo* tests. Hence, in our laboratory, we have planned to investigate the functional role of the variants by creating an *in vitro* model of cells secreting each variant. By transduction, we have already obtained stable cell lines, each expressing a different A-SAA variant, that could be used to stimulate primary human fibroblast-like synoviocytes (data not shown).

On the other hand, the increase of S100A8/S100A9 expression was detected in ERA but there was no decrease after 1-year treatment. Therefore, we proposed that, even though patients can be considered in remission according to the DAS28-CRP, some inflammatory markers can still remain. In this perspective, it is important to stress that we want to go beyond the concept of considering these proteins as biomarkers. Indeed, a larger cohort of patients would have been necessary for a clinical study with a diagnostic purpose. We rather suggest in this preclinical study SAA2α, S100A8, and S100A9 as potential companion markers that should be validated on new cohorts. These could be used for a better follow-up of the disease state fostering higher chance to achieve remission and preserve joint functionality in regard to a longer delay in assessment.

We acknowledged some limitations to our work. Firstly, the studied ERA population reflects a real-life clinical setting, and thus has non-standardized treatment protocol: this has led to a reduction in the sample size for subgroup analysis. Even if the aim of the work was not to find diagnostic biomarkers, we should foresee to select a cohort of patients adhering to a predefined treatment protocol in order to achieve wider consistent statistical analysis, and to monitor quantitative differences for these companion markers at different times. Moreover, it should be interesting to dose these proteins with a shorter and longer timing in order to define more precisely a cut-off of value that could suggest a relapse of the disease and/or the presence of subclinical inflammation.

On the other hand, the study has the strength of using ERA cohort naïve to therapy proposing companion markers which could overcome the limits of DAS28-CRP in detecting subclinical inflammation. In addition, our observations indicate that the assessment of these companion markers can help to better appraise RA activity and identify patients needing a more intensive biologic therapy, before the appearance of structural damage. Probably, the common use of methotrexate as first choice treatment may be rather accompanied by a prompter use of biologic drugs.

## Data Availability Statement

The raw data supporting the conclusions of this article will be made available by the authors, without undue reservation.

## Ethics Statement

The studies involving human participants were reviewed and approved by Ethics Committee of the Cliniques Universitaires Saint-Luc. Written informed consent to participate in this study was provided by the participants’ legal guardian/next of kin.

## Author Contributions

FC performed ELISA and statistical analysis and drew figures. FC and DDS drafted the manuscript. GN, GC, and MF performed the MS analysis. VB, SDR, PS, PD, and TS contributed to collect samples and carried out the clinical evaluation of patients. PD, MF, and MM critically revised the manuscript. MM and DS designed the study, contributed to the interpretation of data, and coordinated the research. All authors contributed to the article and approved the submitted version.

## Funding

This work was supported by CAP48 (RTBF) *via* the arthritis medical research project.

## Conflict of Interest

The authors declare that the research was conducted in the absence of any commercial or financial relationships that could be construed as a potential conflict of interest.

## Publisher’s Note

All claims expressed in this article are solely those of the authors and do not necessarily represent those of their affiliated organizations, or those of the publisher, the editors and the reviewers. Any product that may be evaluated in this article, or claim that may be made by its manufacturer, is not guaranteed or endorsed by the publisher.
