# Supplementary material for: A Cross-Sectional and Longitudinal Study to Define Alarmins and A-SAA Variants as Companion Markers in Early Rheumatoid Arthritis
Source: Front Immunol. 2021 Aug 20;12:638814. doi: 10.3389/fimmu.2021.638814 (PMC8418532; doi:10.3389/fimmu.2021.638814)
Supplement: Supplementary file 1 [file DataSheet_1.pdf]

Supplementary Data 1. Sequences of the five different SAA variants. Each protein is made of 122 aminoacids (aa), the first 18 aa (in italic) constitute the signal peptide. The differences are in bold. SAA1 variants differ from those of SAA2 for aa 15 (in the signal peptide),78, 86, 87, 102, 108. The differences among SAA1α and SAA1β reside in aa 70 and 75; SAA1α differ from SAA1γ for aa 70 while SAA1β and SAA1γ sequences change at aa 75. The difference among SAA2 variants is only at aa 89.

1) SAA1α

15

*MKLLTGLVFC SLVL**G**VSS*RS FFSFLGEAFD GARDMWRAYS DMREANYIGS DKYFHARGNY

DAAKRGPGG**V** WAAE**A**IS**D**AR ENIQR**FF**G**H**G AEDSLADQAA N**E**WGRSG**K**DP NHFRPAGLPE KY

70 75 78 86 87 89 102 108

2) SAA1β

*MKLLTGLVFC SLVL**G**VSS*RS FFSFLGEAFD GARDMWRAYS DMREANYIGS DKYFHARGNY

DAAKRGPGG**A** WAAE**V**IS**D**AR ENIQR**FF**G**H**G AEDSLADQAA N**E**WGRSG**K**DP NHFRPAGLPE KY

3) SAA1γ

*MKLLTGLVFC SLVL**G**VSS*RS FFSFLGEAFD GARDMWRAYS DMREANYIGS DKYFHARGNY

DAAKRGPGG**A** WAAE**A**IS**D**AR ENIQR**FF**G**H**G AEDSLADQAA N**E**WGRSG**K**DP NHFRPAGLPE KY

4) SAA2α

*MKLLTGLVFC SLVL**S**VSS*RS FFSFLGEAFD GARDMWRAYS DMREANYIGS DKYFHARGNY

DAAKRGPGG**A** WAAE**V**IS**N**AR ENIQR**LT**G**H**G AEDSLADQAA N**K**WGRSG**R**DP NHFRPAGLPE KY

5) SAA2β

*MKLLTGLVFC SLVL**S**VSS*RS FFSFLGEAFD GARDMWRAYS DMREANYIGS DKYFHARGNY

DAAKRGPGG**A** WAAE**V**IS**N**AR ENIQR**LT**G**R**G AEDSLADQAA N**K**WGRSG**R**DP NHFRPAGLPE KY
