# Supplementary material for: A Cross-Sectional and Longitudinal Study to Define Alarmins and A-SAA Variants as Companion Markers in Early Rheumatoid Arthritis
Source: Front Immunol. 2021 Aug 20;12:638814. doi: 10.3389/fimmu.2021.638814 (PMC8418532; doi:10.3389/fimmu.2021.638814)

Supplementary Figure 1. Histograms representing the proportion of A-SAA variant. **(A)** Histograms representing the proportion of each A-SAA variant; in the graph on the right ERA patients are classified as good (R) and non (NR) responders. **(B)** Histograms representing the proportion of SAA1α, SAA1β and SAA2α, that are the 3 A-SAA isoforms whose expression changed in pathological condition. In the graph on the right ERA patients are classified as good (R) and non (NR) responders

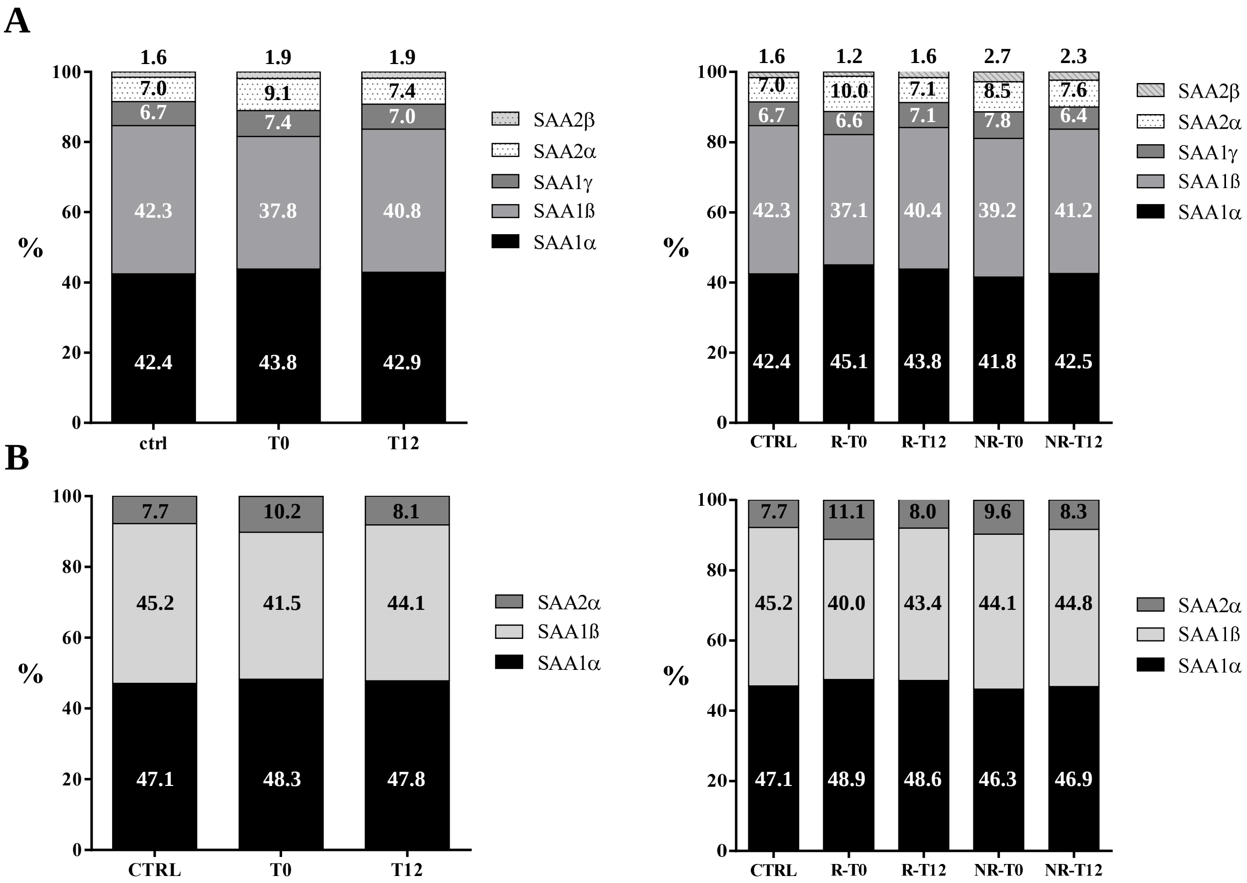

Supplement: Supplementary file 2 [file Image_1.pdf]
