# Supplementary material for: A Cross-Sectional and Longitudinal Study to Define Alarmins and A-SAA Variants as Companion Markers in Early Rheumatoid Arthritis
Source: Front Immunol. 2021 Aug 20;12:638814. doi: 10.3389/fimmu.2021.638814 (PMC8418532; doi:10.3389/fimmu.2021.638814)

Supplementary Figure 2. Correlation among alarmins. **(A)** Correlation between S100A8 and S100A9 in Controls (CTRL) and ERA patients at time T0. **(B)** Correlation between S100A8 and calprotectin in CTRL and ERA patients at time T0. **(C)** Correlation between S100A9 and calprotectin in CTRL and ERA patients at time T0. The Spearman's rank correlation coefficient (r^2) was calculated.

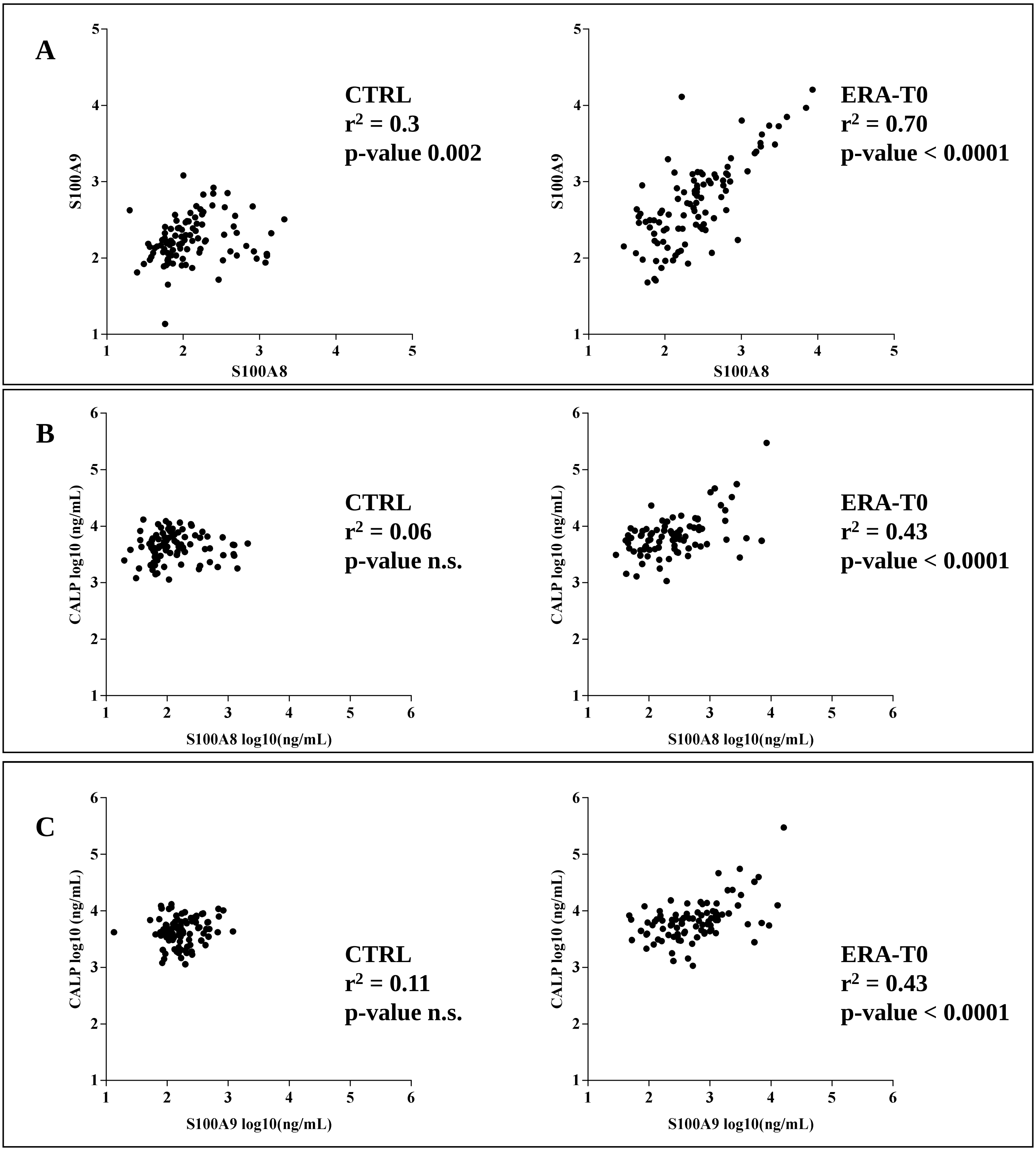

Supplement: Supplementary file 3 [file Image_2.pdf]
