# Supplementary material for: A Cross-Sectional and Longitudinal Study to Define Alarmins and A-SAA Variants as Companion Markers in Early Rheumatoid Arthritis
Source: Front Immunol. 2021 Aug 20;12:638814. doi: 10.3389/fimmu.2021.638814 (PMC8418532; doi:10.3389/fimmu.2021.638814)
Supplement: Supplementary file 4 [file Image_3.pdf]

Supplementary Figure 3. The combinations of proteins giving the better ROC curves in the comparison controls *vs* ERA patients. **(A)** The graph depicts in red the best ROC curve for A-SAA which derived from the combination SAA2α/CRP. For comparison, also the single curves are drawn, together with the curve plotted from ELISA results for total A-SAA. **(B)** The graph depicts in red the best ROC curve for alarmins which derived from the combination S100A8/S100A9/CRP. For comparison, also the single curves are drawn, together with the curve plotted from ELISA results for calprotectin. **(C)** Intrinsic parameters of ROC curves A and B depicted above. AUC area under the curve; PPV positive predictive value; NPV negative predictive value

A

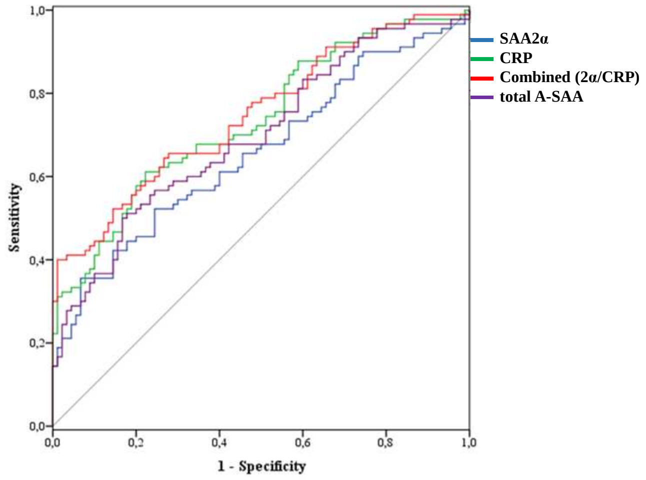

B

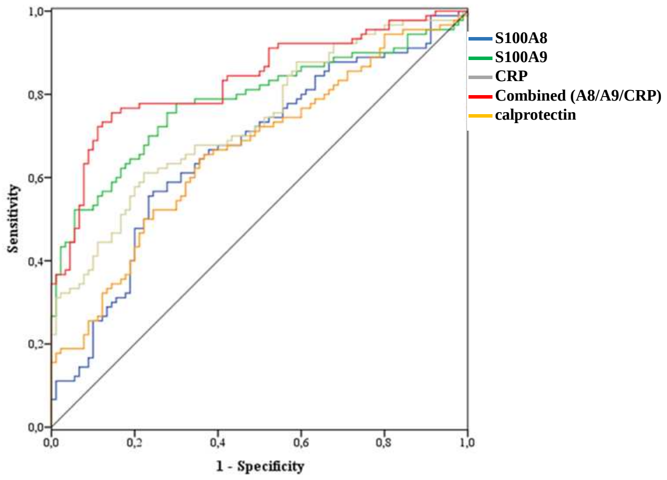

C

	Cut-off	AUC	Sensitivity %	Specificity %	PPV %	NPV %
			A-SAA			
**SAA2α**	0.132 μg/mL	0.657	52.2	74.4	67.1	60.9
**CRP**	1.736 μg/mL	0.735	61.1	77.8	73.3	66.7
**Combined *(SAA2α/CRP)***	***0.458***	***0.748***	***62.2***	***75.6***	***70.2***	***67.7***
**Total A-SAA: ELISA**	25.35 μg/mL	0.698	60.0	67.8	65.0	62.9
			ALARMINS			
**S100A8**	0.199 μg/mL	0.667	55.6	76.7	70.4	63.3
**S100A9**	0.307 μg/mL	0.782	64.4	81.1	77.3	69.5
**CRP**	1.736 μg/mL	0.735	61.1	77.8	73.3	66.7
**Combined *(S100A8/S100A9/CRP)***	***0.455***	***0.834***	***75.6***	***85.6***	***83.9***	***77.8***
**Calprotectin: ELISA**	5.512 μg/mL	0.665	64.4	64.4	64.4	64.4
