# Supplementary material for: A Cross-Sectional and Longitudinal Study to Define Alarmins and A-SAA Variants as Companion Markers in Early Rheumatoid Arthritis
Source: Front Immunol. 2021 Aug 20;12:638814. doi: 10.3389/fimmu.2021.638814 (PMC8418532; doi:10.3389/fimmu.2021.638814)
Supplement: Supplementary file 5 [file Image_4.pdf]

Supplementary Figure 4. Receiver operating characteristic (ROC) curves obtained from the comparison control *vs* ERA patients. **(A)** The graph depicts in red the ROC curve which derived from the combination SAA1α/SAA1β/SAA1γ/SAA2α/SAA2β. For comparison, also the single curves are drawn, together with the curve plotted from ELISA results for total A-SAA. **(B)** The graph depicts in red the ROC curve which derived from the combination SAA2α/CRP/total A-SAA. For comparison, also the single curves are drawn. **(C)** The graph depicts in red the ROC curve which derived from the combination total A-SAA/CRP. For comparison, also the single curves are drawn.

A

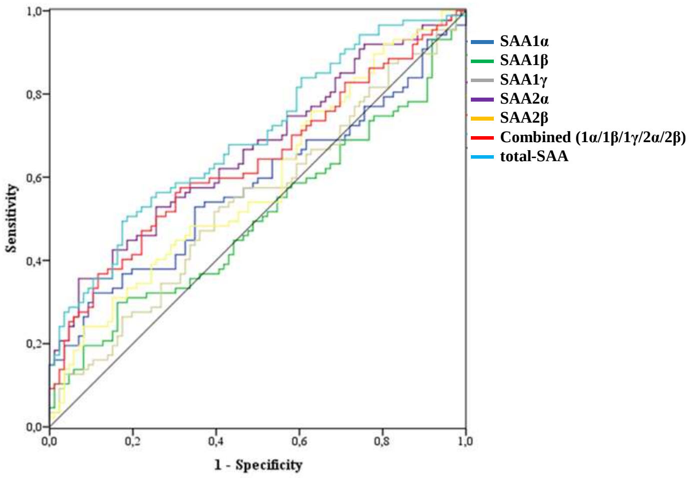

	AUC	Sensitivity %	Specificity %
**SAA1α**	0.578	54	62.8
**SAA1β**	0.502	31	81.4
**SAA1γ**	0.543	51.7	60.5
**SAA2α**	0.657	52.2	74.4
**SAA2β**	0.586	44.8	69.8
**Combined**	0.635	56.3	69.8
**Total A-SAA: ELISA**	0.698	60	67.8

B

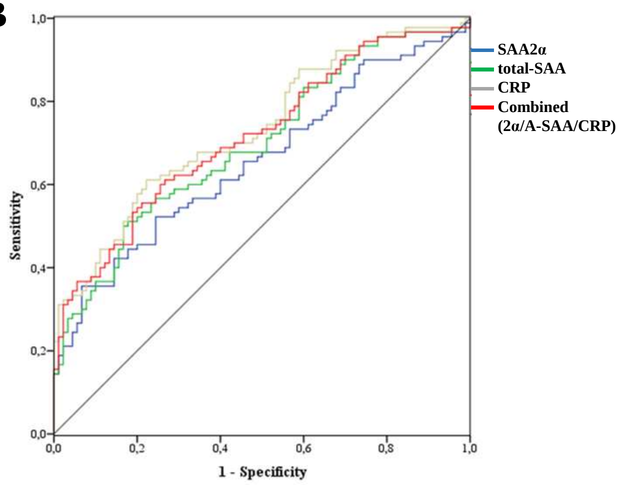

	AUC	Sensitivity %	Specificity %
**SAA2α**	0.657	52.2	74.4
**Total A-SAA: ELISA**	0.698	60	67.8
**CRP**	0.735	61.1	77.8
**Combined**	0.718	60	74.4

C

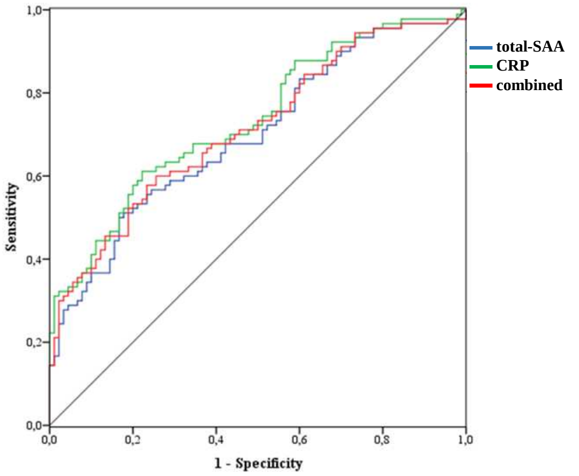

	AUC	Sensitivity %	Specificity %
**Total A-SAA: ELISA**	0.698	60	67.8
**CRP**	0.735	61.1	77.8
**Combined**	0.713	60	74.4
