# Supplementary material for: A Cross-Sectional and Longitudinal Study to Define Alarmins and A-SAA Variants as Companion Markers in Early Rheumatoid Arthritis
Source: Front Immunol. 2021 Aug 20;12:638814. doi: 10.3389/fimmu.2021.638814 (PMC8418532; doi:10.3389/fimmu.2021.638814)

Supplementary Figure 5. Differences in the expression of proteins in patients sorted by treatment. **(A)** Differences in the expression of CRP in patients, at T0 and T12, sorted by treatment: methotrexate monotherapy (MTX) or biologics (BIO). **(B)** The expression of total A-SAA in patients, at T0 and T12, sorted by treatment. R, good responders; NR, non responders. Scatter dot plots represent the median with interquartile range. * p-value ≤ 0.05, ** p-value ≤ 0.01, *** p-value ≤ 0.001 (Wilcoxon test).

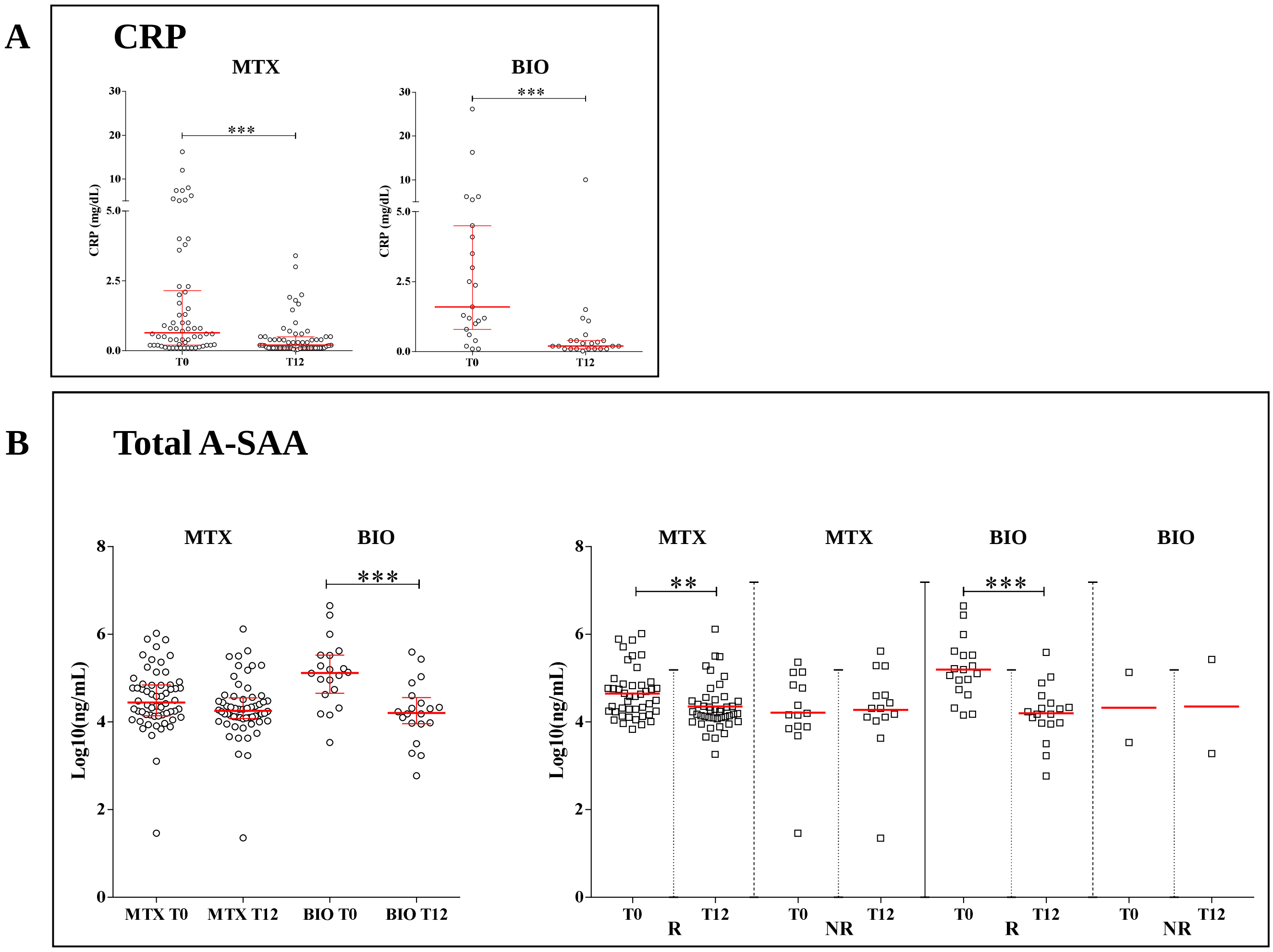

Supplement: Supplementary file 6 [file Image_5.pdf]
